# Exploring the Impact and Path of Environmental Protection Tax on Different Air Pollutant Emissions

**DOI:** 10.3390/ijerph19084767

**Published:** 2022-04-14

**Authors:** Weijiang Liu, Tingting Liu

**Affiliations:** 1 Center for Quantitative Economics, Jilin University, Changchun 130012, China; liuwj@jlu.edu.cn; 2 School of Business and Management, Jilin University, Changchun 130012, China

**Keywords:** environmental protection tax, threshold effect, mediating effect, pollutant emissions

## Abstract

Existing studies have examined the double dividend effect of environmental protection tax. However, less attention has been paid to the influencing factors and transmission paths of the pollution abatement effect of the environmental protection tax. Based on the panel data for 30 of China’s provinces from 2007 to 2019, this study discusses the environmental protection tax’s influencing factors and transmission paths on the emission scale and intensity of different air pollutants through the panel threshold regression model and mediating effect model. The results show that: (1) the environmental protection tax has a positive emission reduction effect on the emission scale or emission intensity of sulfur dioxide (SO_2_) and nitrogen oxides (NO_2_); (2) the abatement effect is stronger when per capita gross regional product is above the threshold value; (3) technological progress, economic growth, and industrial structure all have positive mediating effects. Therefore, the local environmental protection tax rate should be set with comprehensive consideration of regional economic development, industrial structure, and technological progress.

## 1. Introduction

Since the reform and opening up in 1978, China’s economic growth rate has been significantly higher than the average world level. However, the ensuing air pollution problem has also aroused widespread concern in the community. Therefore, in 2018, the “Environmental Protection Tax Law of the People’s Republic of China” was implemented, and China began to collect a special environmental protection tax. It is economical to internalize the social costs of environmental pollution and ecological damage into production costs and market prices to reduce pollutant emissions and promote cleaner production [1,2]. However, the air pollution situation has not improved significantly in the short term. In 2019, Total SO_2_ emissions were up to 4.573 million tons, and NO_2_ emissions were 12.339 million tons. Based on the “Statistical Bulletin of the People’s Republic of China on the National Economic and Social Development”, in 2019, among the 31 major cities, only 13 cities had standard air quality for more than 300 days. In 2020, only 59.9% of the 337 cities monitored at prefecture-level and above will have up to standard air quality. Furthermore, continuous air pollution has surpassed the self-purification capacity of the ecological environment, causing air pollution and increasing people’s morbidity [3]. Therefore, to better play the pollution reduction role of environmental protection tax, it is crucial to explore the factors influencing the pollution reduction effect of environmental protection tax. In addition, it is also necessary to further clarify the transmission path of environmental protection tax affecting pollutant emissions.

Current scholars have conducted in-depth research on the double dividend effect of environmental protection tax [4]. Still, most of the research has focused on the optimal taxation method and rate to achieve balanced economic development and environmental protection [5,6,7]. There is less investigation on the factors affecting the pollution reduction effect of environmental protection tax. The imposition of environmental protection taxes will increase production costs for enterprises and costs for consumers. Hence, at different levels of regional economic development, there are differences in people’s requirements for environmental quality, and the technical ability to carry out enterprise pollution reduction also differs, which will directly affect the policy effect of environmental protection tax. In our study, we first constructed a panel threshold model using the per capita domestic production level as the threshold to analyze the emission reduction effect of the environmental protection tax levy on SO_2_ and NO_2_ pollutant emissions. In particular, the mediating effect model is adopted to identify the path mechanism of different pollutants further.

The contributions of this study to the existing literature cover three aspects: (1) research on environmental protection tax has mainly focused on exploring the double dividend: the impact on the relationship between economic growth and pollutant emissions. There is little literature analyzing how environmental protection tax affects the reduction in pollutant emissions and the role of per capita GDP. We explored the nonlinear effects and mediating path of the environmental protection tax on different pollutants using the threshold regression and mediating effect models. Research results enrich the environmental protection tax literature. (2) There have been studies on reducing pollutants by environmental protection tax, and most of them use a pollutant or pollutant synthesis index. However, the taxation policies have different biases and impacts on different pollutants. This paper selects two different pollutants (sulfur dioxide, nitrogen oxides) and two different indicators (emission scale, emission intensity) for comparative analysis of the emission reduction effects and paths of the environmental protection tax on different pollutants. (3) In terms of method, compared with the traditional causal stepwise regression approach proposed by Baron and Kenny [8] and the Sobel test [9] to estimate the mediating effect model, a more sensitive and effective bootstrap mediating effect analysis method is used [10].

This paper is arranged as follows: Section 2 presents the literature review and hypothesis; Section 3 describes the methods and data; Section 4 demonstrates the empirical results and discussion; Section 5 concludes the results, provides recommendations, and notes the study’s limitations and future research direction.

## 2. Literature Review and Hypothesis

### 2.1. Environmental Protection Tax and Pollutants Emission

As the international community has widely recognized the theory of sustainable development, the issue of environmental protection has attracted the attention of governments of all countries. As a direct and effective tax to coordinate the environment and economic development, the environmental protection tax has become the primary method of environmental governance. It has also been a hot issue in recent years.

Currently, the pollutant reduction effect of environmental taxes has been confirmed by a number of existing studies [11,12,13]. Specifically, from a metrological test perspective, Han [14] adopted the Bayesian space–time model to identify the spatial and geographic characteristics of the environmental protection tax on PM_2.5_ pollution, using the Bayesian LASSO regression model to estimate the environmental reduction coefficient is 12.1% and discern the two most significant influencing factors: urbanization rate and relief amplitude. From a tax policy simulation perspective, scholars have explored this in either single tax policy effects or tax combination coordination effects [15]. Zhao [16] applies a dynamic stochastic general equilibrium (DSGE) model to simulate the policy effects of carbon taxes and carbon trading and their combinations, showing that both hurt the economy. Xu [17] used the computable general equilibrium (CGE) model, showing that carbon tax can significantly curb traditional energy consumption and emissions. Combined with the value-added tax reform, it can achieve the double dividend of a carbon tax. A few studies argue for a different view [18]. Fredriksson [19] claims that environmental tax will increase pollutant emissions by reducing net revenue. Tobin [20] stated that environmental tax has no significant impact on reducing the environmental pollution. Although there is no consensus on the pollution abatement effects of environmental protection taxes, this may be due to the choice of emissions and measurement indicators.

Further analysis shows that economic development is closely related to the pollutant emission reduction effect of the environmental protection tax. In regions with different levels of economic development, on the one hand, people have different preferences in economic growth and green environments [21]; on the other hand, green technology research and social responsibility are different [22]. They will affect the policy implementation effect of the environmental protection tax. Moreover, any policy that targets GDP will affect pollutant emissions [23]. Therefore, for environmental protection tax to play a full policy role, economic development must be considered [24,25]. By analyzing the effects of environmental protection in all EU member states, Miceikiene [26] showed that the impacts of environmental tax are stronger in countries with slower economic and tax growth but faster development of renewable energy production technologies. He [27] reached a different conclusion: the larger the scale of economic development, the better the environmental protection tax reduction in OECD counties and Chinese provinces. Hu [28] used a multi-region multisector computable general equilibrium model to study China at the provincial level and came to the same conclusion. The regulation of air pollutant emissions by pollution tax policies only significantly impacts regions with larger economies (e.g., Guangdong, Shandong, and Zhejiang). Furthermore, the ratio of environmental-related tax revenue to GDP exceeds a certain threshold and significantly reduces the ecological deficit [29].

### 2.2. Analysis of the Path of Environmental Protection Tax to Different Pollutants Emissions

**Figure 1:**
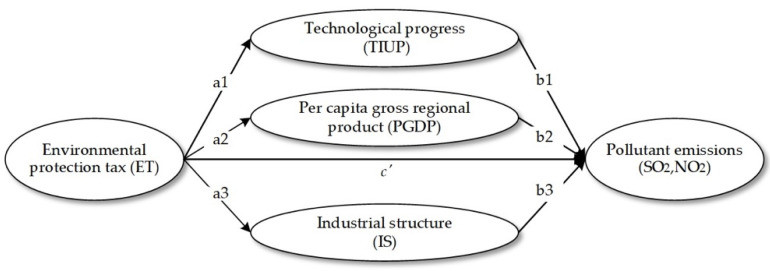
The mediation mechanism path of the environmental protection tax.

To analyze the impact path of the environmental protection tax on pollutant emissions, we must first clarify the influencing factors of pollutant emissions. Concerning Brock [30], from the perspective of production, pollutant emissions are theoretically determined by three paths: namely, the total economic volume, industrial structure, and technological level. Therefore, let Y represent the total GDP output of an economy; $s_{i}$ and $\rho_{i}$ describe the ratio of industry $i^{\prime }s$ GDP to the total output and industry $i^{\prime }s$ pollution emissions per unit GDP, respectively. E denotes the total pollutants emission of an economy, which can be defined by

(1) $$E=\sum_{i=1}^{n}\rho_{i}s_{i}Y_{i}$$

where $\sum_{i=1}^{n}s_{i}=1$. The two sides of Formula 1 are derived concerning time *t*, respectively, and the change of pollution emission is decomposed as Formula (2),

(2) $$\hat{E}=\sum_{i=1}^{n}\pi_{i}({\hat{\rho }}_{i}+{\hat{s}}_{i})+\hat{Y}$$

where $\hat{x}=\frac{dx}{dt}\cdot \frac{1}{x},x=\left\{E,Y,s_{i},\rho_{i}\right\}$ is the relative time change of each variable, and $\pi_{i}=\frac{E_{i}}{E}$ is the proportion of pollutants produced by industry i in the total pollutants. Variables ${\hat{\rho }}_{i}$, ${\hat{s}}_{i}$, and $\hat{Y}$ determine the changes in environmental pollution, and represent the three effects that affect environmental pollution changes: technological progress, economic growth, and industrial structure. Given that the other two factors remain unchanged, technological progress will reduce pollutants per unit of output and promote the decline of pollution levels. Economic growth will increase pollutant emissions. The increase in high-polluting industries will increase pollutant emissions, and the increase in the proportion of low-polluting industries will reduce pollution. However, due to the externality of environmental pollution, the effects of technological progress and industrial structure will not automatically be realized by market regulation [31,32]. To a certain extent, government environmental policy induction is necessary. Environmental protection tax is the most commonly used and directly effective environmental policy. Imposing environmental protection tax can accelerate the advancement of environmental-related technologies to reduce carbon emissions and sustainable development in high-income or middle-income countries [22]. Then, R&D spending is recommended by Fernandez [33] as an engine for economic development and as a driving force for sustainable economic development. Simultaneously, the empirical results also show that R&D expenditure positively reduces CO_2_ emissions, emphasizing the need to strengthen measures to achieve the decoupling between energy consumption and emissions. Fan [21] demonstrates the particular evolution paths of economic growth, pollution intensity, and resource intensity under different environmental protection tax parameters. Results indicate a robust beneficial role of an environmental protection tax on green development. Furthermore, when an environmental protection tax is imposed, firm government control, active consumer awareness, and advanced technology can stimulate economic growth and decrease pollution intensity. Nevertheless, government control has a more substantial effect. From an industrial structure perspective, environmental protection tax increases the cost of production for highly polluting industries, forcing enterprises to improve the energy consumption structure by introducing clean energy and green production methods [34]. This ultimately reduces pollutant emissions, while enterprises transform and upgrade. $c^{\prime }$ is direct effect environmental protection tax on pollutant emissions. The mediation mechanism is shown in Figure 1.

### 2.3. Hypotheses

Therefore, combining the above analysis with the current situation in China, we propose the following research hypotheses:

**Hypothesis** **1.** *Environmental protection tax can reduce pollution emissions;*

**Hypothesis** **2.** *Different levels of economic development have different emission reduction effects* *;*

**Hypothesis** **3.** *Technological progress, economic development, and industrial structure positively mediate the pollution reduction effect of environmental tax.*

## 3. Methods and Data

### 3.1. Models

#### 3.1.1. Panel Threshold Regression Model

As described above, due to the differences in the environmental quality requirements of regions with different economic development levels, the implementation of environmental tax has different effects on pollutant emissions. Hence, we use the threshold regression model to explore the impact of environmental protection tax on pollutants emission scale and intensity, which identifies regression at different stages through thresholds. In this article, pollutants emission (*PE*) mainly refers to sulfur dioxide emission and nitrogen oxides emission. They are measured through emission scale (SO_2,_ NO_2_) and emission intensity (GSO_2_, GNO_2_), respectively, as dependent variables. Environmental protection tax (*ET*) is taken as an independent variable. Per capita gross regional product (PGDP) is regarded as a threshold variable to test whether there is a threshold effect on the relationship between environmental protection tax and pollutants emission. Therefore, this paper adopts the Hansen panel threshold model to construct a single threshold model as Formula (3)

(3) $$\begin{array}{c} PE_{it}=\alpha_{1}ET_{it}\cdot I(PGDP_{it}\leq \delta )+\alpha_{2}ET_{it}\cdot I(PGDP_{it}>\delta )\\ +TIUP_{it}+PGDP_{it}+ISU_{it}+PCP_{it}+FSS_{it}+\varepsilon_{it} \end{array}$$

where, i and t denote province and year, respectively; PE refers to the emission scale (SO_2_, NO_2_) and emission intensity (GSO_2_, GNO_2_); TIUP denotes technological progress; IS denotes Industrial structure; PCP and FSS are per capita park green area and fiscal self-sufficiency rate, respectively. δ is the threshold to be estimated; I(⋅) is the index function, and the value is 1 when the conditions in the brackets are satisfied; otherwise, it is 0. ε is the random disturbance term. α are the parameters to be estimated. Based on the single threshold model, a double threshold model can be built as Formula (4),

(4) $$\begin{array}{l} PE_{it}=\alpha_{1}ET_{it}\cdot I(PGDP_{it}\leq \delta_{1})+\alpha_{2}ET_{it}\cdot I(\delta_{1}<PGDP_{it}\leq \delta_{2})\\ +\alpha_{3}ET_{it}\cdot I(PGDP_{it}>\delta_{2})+TIUP_{it}+PGDP_{it}+ISU_{it}+PCP_{it}+FSS_{it}+\varepsilon_{it} \end{array}$$

#### 3.1.2. Mediating Effect Model

The mediating effect model is conducive to exploring the internal effect path of the independent variable on the dependent variable [35]. Generally speaking, we analyze the specific influence of the independent variable X on the dependent variable Y. If variable X influences variable Y by affecting the variable M, then the variable M is called the mediating variable. The role of the mediating variable M is called the mediating effect. In a simple mediating model, the analysis of mediating effect is shown as follows:

(5) $$Y=cX+e_{1}$$

(6) $$M=aX+e_{2}$$

(7) $$M=aX+e_{2}$$

where, *c* is the main effect, that is, the total effect of X on Y; a is the effect of X on M; b is the effect of M on Y when the influence of X is controlled; $c^{\prime }$ is the direct effect of X on Y when the influence of M is controlled; $e_{1},e_{2}$ and $e_{3}$ are random disturbance terms. Here, the mediating effect is equal to the indirect effect (ab). The quantitative relationship among total effect (c), direct effect ($c^{\prime }$), and indirect effect (ab) can be expressed as Formula (8) (Mackinnon et al., 1995) [36]:

(8) $$c=c^{\prime }+ab$$

Combining the above analysis and specific variables used in this paper, Y denotes the pollutant emissions; X refers to the environmental protection tax; M consists of TIUP, PGDP, and IS, assuming there is no interaction among them.

The causal step approach proposed by Baron and Kenny [8] is commonly used to estimate the mediating effect model. When the regression coefficient meets the following conditions simultaneously, the mediating effect exists. Both a and b are statistically significant and $c^{\prime }$ is not significant, or the effect is significantly reduced relative to c. However, in recent years, many works of literature have questioned the method of the causal step approach, regardless of the validity of the test method or the rationality of the test procedure [37,38]. Therefore, in our paper, we refer to Zhao [10], using the bootstrap approach proposed by Preacher [9] to test the mediating effect.

### 3.2. Variables

#### 3.2.1. Dependent Variable

Dependent variables include the sulfur dioxide emission scale (SO_2_), nitrogen oxides emission scale (NO_2_), sulfur dioxide emission intensity (GSO_2_), and nitrogen oxides emission intensity (GNO_2_). SO_2_ and NO_2_ are the primary air pollutant emissions and the leading cause of acid rain. It is more closely related to the ecological environment and human health (such as increasing the incidence of respiratory infections). Therefore, two pollutants, SO_2_ and NO_2_, are selected in this paper and measured from two indicators of emission scale and intensity. The emission scale is the total amount of SO_2_ and NO_2_ emissions in the industrial production process; the emission intensity is measured by the ratio of the emission scale to the GDP deflated by the GDP deflator.

#### 3.2.2. Independent Variable

Environmental protection tax (ET) is the independent variable. The connotation of environmental protection tax has two scopes, a narrow sense and a broad sense. The broad definition of environmental protection tax is the most widely used among them. Since China has only enacted the special environmental protection tax since 2018 and the availability of data is thus limited, this paper uses the broad environmental protection tax to measure environmental protection tax under the OECD’s definition of environmental protection tax. All taxes and fees collected by the government are compulsory, free of charge, and related to environmental protection. Mainly including resource tax, fixed asset investment direction adjustment tax, urban maintenance and construction tax, urban land use tax, vehicle and vessel use license tax, cultivated land occupation tax, pollution discharge fee, and environmental protection tax. In order to enhance the robustness of data, we use the ratio of broad environmental protection tax to total tax to measure environmental protection tax [39].

#### 3.2.3. Mediating Variable

The first mediating variable is technological progress (TIUP). The environmental protection tax has a double impact on technological progress. Levying environmental protection tax will increase the production costs of enterprises and encourage enterprises to carry out technological innovation. However, the excessive environmental protection tax will squeeze out enterprises’ R&D investment, which is not conducive to technological progress. The impact of technological progress on pollutant emissions is also a double-edged sword. Technological progress optimizes energy consumption: improving the efficiency of fossil energy use and the proportion of clean energy use will help reduce pollutant emissions. Nevertheless, at the same time, technological progress will reduce the cost and price of a unit product, leading to more production and consumption and thereby increasing emissions. Our paper refers to Huang [40] to measure technological progress using invention and utility patents.

The second mediating variable is per capita gross regional product (PGDP). There has been no consensus on the relationship between economic growth and environmental pollution. It is commonly described by the environmental Kuznets curve (EKC) [41]. Some scholars use the EKC theory to empirically test the inverted U-curve relationship between economic growth and environmental pollution [42,43]. Other scholars have also empirically proved the existence of the N-shaped curve [44] and the M-shaped curve. Different regions have different levels of economic development, and the environmental protection tax has different emission reduction effects. In regions with high economic development levels, people have a higher demand for environmental quality. They have more economic strength to carry out technological innovations. The environmental tax has a more significant effect on emission reduction. Therefore, PGDP is an appropriate variable to measure the threshold effect of environmental protection tax on pollutant emissions.

The third mediating variable is industrial structure (IS). On the one hand, environmental protection tax will increase fossil energy use costs and optimize the industry’s energy consumption structure. On the other hand, it will increase the production costs of high-polluting industries and encourage enterprises to transform and upgrade. Therefore, the collection of environmental protection tax affects pollutant emissions through the adjustment of industrial structure. The industrial structure is measured by ratio; namely, the added value of the tertiary industry is divided by the added value of the secondary industry [45].

#### 3.2.4. Control Variable

Per capita park green area (PCP) is a control variable. Plants can effectively reduce gas pollution and absorb harmful gases. Green space is an essential part of ecological environment construction and protection [46].

Fiscal self-sufficiency rate (FSS) is another control variable. It is a measure of the extent to which a region is financially self-reliant and reflects the autonomy of local governments in environmental governance. Referring to Liu’s fiscal science and technology expenditure [47], we measure the fiscal self-sufficiency rate using the ratio of local fiscal revenue to local fiscal expenditure.

### 3.3. Data

The panel data used in this article are constituted of China’s 30 provincial-level regions between 2007 and 2019. Tibet, Taiwan, Hong Kong, and Macau are excluded because of data unavailability. Pollutants discharge data and environmental protection tax stem from the *China Statistical Yearbook on Environment*. Besides, the number of invention patents and utility patents, per capita gross regional product, the added value of the second industry, and the tertiary industry are derived from the *China Statistical Yearbook*. Per capita park green area and data used to calculate fiscal self-sufficiency rate come from the statistical yearbooks of various provincial-level regions. In addition, the raw data are deflated by the 2007 constant price index. Table 1 lists the definitions and descriptions of variables; the statistical description of variables is presented in Table 2.

## 4. Results and Discussion

### 4.1. Threshold Effect of Environment Protection Tax on Pollutants Emission

Table 3 reports the model specification test of the two pollutants from emission scale and emission intensity: SO_2_, NO_2_, GSO_2_, and GNO_2_, respectively.

**Figure 2:**
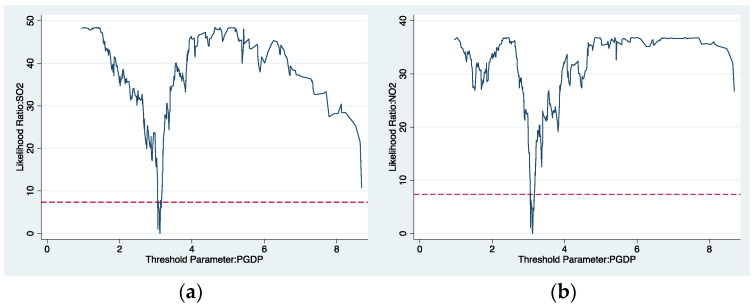

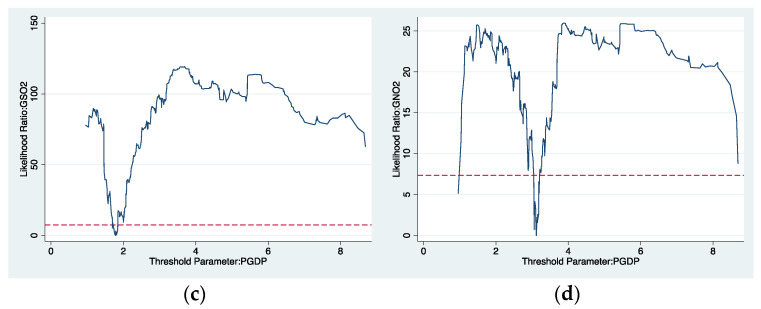
LR function graph of threshold variables: (**a**) SO_2_; (**b**) NO_2_; (**c**) GSO_2_; (**d**) GNO_2_.

As shown in Table 3, the single threshold of all dependent variables is statistically significant at 1%, 5%, and 10% levels, respectively. In contrast, the double and triple thresholds are not all significant. Figure 2a–d shows the likelihood ratio (LR) functions of threshold variables. Combining the threshold significance and LR function graph, we chose the single threshold for analysis. The threshold regression results are shown in Table 4. It is not difficult to find that environmental protection tax has a pronounced emission reduction effect on emission scale or intensity.

#### 4.1.1. Threshold Effect of Environmental Protection Tax on SO_2_ and NO_2_

As mentioned above, the impact of an environmental protection tax on the pollutant emissions scale depends on the level of PGDP. According to Models (1)–(4) in Table 3 and Table 4, environmental protection tax has a weaker abatement effect on SO_2_ and NO_2_ when PGDP is less than the threshold value of 3.116 (first stage). Such an emission reduction effect is more remarkable when PGDP is greater than 3.116 (second stage). The regression results indicate that ET exerts an abatement effect on both SO_2_ and NO_2_. Furthermore, the reduction effect of SO_2_ is greater than that of NO_2_. The higher the PGDP, the stronger the emission reduction effect of ET.

This result is consistent with Hypothesis 1 and previous studies [48], which can be explained from the following perspectives. First, on the one hand, collecting environmental tax will increase production costs and encourage enterprises to innovate in technology or optimize energy input, which is conducive to pollutant emission reduction. On the other hand, the increase in cost will lead to large-scale production of some enterprises to obtain scale benefits and then increase pollution emissions. When the first effect is greater than the second effect, the environmental protection tax reduces emissions. Second, in recent years, the acid rain caused by SO_2_ has been subject to control in China, such as the two-control zone policy and SO_2_ emission rights trading pilots. The difference in policy emphasis has led to the difference in the role of environmental tax in reducing emissions of different pollutants. Third, areas with high-income levels have a higher demand for green ecology and green products. People have more vital environmental protection awareness, which is conducive to implementing environmental protection tax policies. At the same time, companies have the economic strength to carry out green technology innovation research and development instead of relying on scale economy to reduce costs.

Concerning the mediating variables in Models (1)–(4), the coefficients of TIUP and ISU are significantly negative at the 1% level. The coefficients of PGDP are positive. In terms of control variables, PCP is negative (−2.608), and FSS is positive (0.776) in Model 3; they are not significant in Model 4. The impact of technological progress, economic growth, and industrial structure on pollutants emission scale are consistent with expectations. The effects of technological progress and industrial structure on SO_2_ and NO_2_ emissions are negative at a significant level of 1%, and technological progress has a more significant emission reduction effect. PGDP is significantly positive for the pollutant emissions scale, but its coefficient for NO_2_ is 7.872, which is significantly greater than 3.581 for SO_2_.

#### 4.1.2. Threshold Effect of Environmental Protection Tax on GSO_2_ and GNO_2_

According to Models (5)–(8) in Table 3 and Table 4, the PGDP threshold for the impact of an environmental protection tax on GSO_2_ is 1.782, and GNO_2_ remains at 3.116. Similar to the emission scale, the environmental protection tax reduces pollutants’ emission intensity. Next, we analyze the results specifically. In Models (5)–(6), BET coefficients are −4.603 and −3.248 when PGDP is greater than the threshold. Its absolute value is much greater than when PGDP is less than the threshold. Regarding the impact of an environmental protection tax on GNO_2_ in Models (7) and (8), the emission reduction effect of the second stage is greater than that of the first stage. That is, in economic development areas, environmental protection tax has greater emission reduction intensity. This result is in line with expectation and is consistent with Hypothesis 2. Unlike the emissions scale, the environmental protection tax has a more significant effect on GNO_2_ than GSO_2_ in the first stage and the opposite in the second stage. We believe this is related to environmental supervision and policy implementation in economically developed regions. Besides, whether in the first stage or the second stage, the effect of an environmental protection tax on the intensity of pollutants emission is greater than that of the pollutant emissions scale.

Except for the TIUP and PGDP, the results of variables ISU, PCP, and FSS when dependent variables are GSO_2_ and GNO_2_ are similar to those when dependent variables are SO_2_ and NO_2_. The effects of variables TIUP and PGDP on GNO_2_ were not significant. Variable TIUP positively impacts GSO_2_, which is not in line with expectations. We guess that many types of technological progress are divided into production-based technological advancement and green technological advancement. Production-oriented technological progress will promote enterprise production and increase pollution emissions. Green technological progress will reduce pollution emissions. When the increasing effect is higher than the decreasing effect, it will cause the pollution emission intensity to increase Yao [49]. Variable PGDP exerts a negative effect on GSO_2_, which is in accordance with our expectations.

### 4.2. Mediating Effect of Environmental Protection Tax on Pollutants Emission

As mentioned above, there are doubts about the rationality of the distribution regression method to test the mediating effect, and there may be a masking effect among the mediating variables. In order to more clearly identify the path of the environmental protection tax on pollutant emissions, we use the bootstrap method to directly test the mediation effect “ab” for mediation effect analysis. The empirical results of the mediating effect of the environmental protection tax on pollution emission scale and intensity are consistent with Hypothesis 3.

#### 4.2.1. Mediating Effect of Environmental Protection Tax on SO_2_ and NO_2_

As shown in Table 5, the total effect coefficient c of the environmental protection tax on SO_2_ is −3.188 with a 1% significance level. However, when the intermediate variables are TIUP, PGDP, and ISU, the corresponding direct coefficients C^′^ are −2.799, −1.296, and −6.687, at the 1%, 5%, and 1% significance levels, respectively. The above indicates that both TIUP, PGDP, and ISU all have a mediating effect on SO_2_. According to the bootstrap test, the mediation effect coefficients ab of TIUP, PGDP, and ISU are −0.389, −1.892, and −1.130, respectively, with a 1% significance level. Among them, the mediating effect of PGDP is the largest, accounting for about 59.3% of the total effect. The mediating effect of an environmental tax on NO_2_ is similar to that of SO_2_, but ISU is the most prominent mediator. Specifically, the total effect coefficient c of an environmental protection tax on NO_2_ is −1.403 with a 5% significance level. The mediation effect coefficients ab of TIUP, PGDP, and ISU on NO_2_ are −0.238, −0.152, and −0.911, respectively, with a 1% significance level. Based on the above analysis, we found that although TIUP, PGDP, and ISU have mediation effects on SO_2_ and NO_2_, the effect is different for different pollutants.

#### 4.2.2. Mediating Effect of Environmental Protection Tax on GSO_2_ and GNO_2_

According to the bootstrap mediation effect test results in Table 6, TIUP, PGDP, and ISU negatively affect GSO_2_ with 1% and 5% significance levels. The coefficient of TIUP is −0.245, which means that each 1% increase in BET can cause a 0.245% decrease in GNO_2_ because of BET’s effect on TIUP. The coefficients of PGDP and ISU are −2.434 and −1.172, respectively. The coefficients of direct effect are −7.613, −5.424, and −6.687, respectively, with a 1% significance level. Among them, the variable PGDP has the most significant mediating effect, accounting for 31% of the total effect. The total effect of the environmental protection tax on GNO_2_ is −4.328 with a 1% significance level. The indirect effects of TIUP, PGDP, and ISU are −0.155, −1.112, and −0.752 with 5%, 1%, 10% significance levels, respectively. This shows that the mediating variable has a mediating effect on GNO_2_, and the direction of action is in line with expectations. For example, each 1% increase in BET can cause a 1.12% decrease in GNO_2_ because environmental protection tax acts on PGDP. Similar to the effect of PGDP on GSO_2_, PGDP has the most significant mediating effect on GNO_2_, accounting for about 25.7% of the total effect.

## 5. Conclusions

In order to give full play to the pollution abatement effect of environmental protection tax, based on the panel data of 30 provinces in China from 2007 to 2019, we use the emissions of SO_2_ and NO_2_ from two indicators: emissions scale and emissions intensity as the dependent variables for a more comprehensive analysis. The panel threshold regression model was implemented to study the causal relationship between environmental protection tax and pollutant emissions, with per capita GDP as the threshold variable. The mediation effect model explored the impact path mechanism of the environmental protection tax on pollutant emissions.

From the above result analysis, we draw the following conclusions. Firstly, the levy of the environmental protection tax is indeed conducive to reducing SO_2_ and NO_2_ emissions scale and emissions intensity. Overall, regarding emissions scale, the environmental protection tax has a greater effect on SO_2_ than NO_2_. However, in terms of emissions intensity, the effect of NO_2_ is greater than that of SO_2_. More importantly, the emission reduction effect of environmental protection taxes is significantly enhanced when per capita GDP is above the threshold, both in terms of emissions scale and emissions intensity regarding SO_2_ and NO_2_. That is to say, areas with high levels of economic development are more conducive to achieving the effects of environmental protection tax policies. Secondly, the relationships between the environmental protection tax and pollutants SO_2_ and NO_2_, both in terms of emissions scale and emission intensity, are positively mediated by three mediating variables, i.e., technological progress, economic growth, and industrial structure. However, for different pollutants, the main conduction pathways are different. Specifically, in terms of SO_2_ emissions and emission intensity, per capita GDP has the most substantial mediating effect, followed by industrial structure. In terms of NO_2_ emissions, the industrial structure has the most significant mediating effect, and per capita GDP has the strongest mediating effect on GNO_2_.

Based on the above conclusions, corresponding policy revelations can be inferred. Firstly, PGDP is above the threshold and the pollution abatement effect of the environmental protection tax is stronger. Economic development and environmental protection are mutually reinforcing, and a win–win relationship exists. Secondly, China’s government should implement policies to facilitate the optimization of industrial structure, technological progress, and the increase in PGDP. This is because all three mediating variables positively moderate the reducing effects of environmental protection tax policies. Luckily, we are encouraged to see that China is making headway towards this goal. For one thing, China has implemented a policy of ‘two control zones’ and emissions trading, raising the cost of pollutant emissions and forcing technological progress and industrial structure upgrading. For another, energy-use rights trading has been piloted in four provinces (Zhejiang, Fujian, Henan, and Sichuan) and has achieved good results in improving the energy consumption structure. Lastly, all three mediating variables have a positive moderating effect, indicating a synergistic effect of pollutant treatment. Furthermore, the intensity of the mediating pathway varies from pollutant to pollutant and should be tailored to the specific pollutant.

This paper explores the causal relationship and impact path of the environmental protection tax on pollutant emissions at the provincial level, supplementing environmental protection tax and pollutants research. However, given the availability of detailed data, our article has certain limitations. In the real economy, many factors affect the emission of pollutants as aa result of implementing environmental protection tax. Future research will add more mediating variables for in-depth exploration. In addition, a more detailed classification of technological progress is carried out to study the effects of different types of technological progress.

## Figures and Tables

**Table 1:** Definitions and descriptions of variables.

	Variable	Meaning	Index or Source
Dependent variable	SO_2_	Sulfur dioxide emissions scale	Volume of sulfur dioxide emissions
	NO_2_	Nitrogen oxide emissions scale	Volume of nitrogen dioxide emissions
	GSO_2_	Sulfur dioxide emissions intensity	Emissions per unit GDP
	GNO_2_	Nitrogen oxides emissions intensity	Emissions per unit GDP
Independent variable	BET	Environmental protection tax	*China Statistical Yearbook on Environment*
Mediating variable	TIUP	Technological progress	Total of invention and utility patents
	PGDP	Per capita GDP	Per capita GDP
	IS	Industrial structure	Ratio of added value of tertiary industry to secondary industry
Control variable	PCP	Per capita park green area	Per capita park green area
	FSS	Fiscal self-sufficiency rate	Ratio of budgeted expenditure/budgeted income

**Table 2:** Statistical description of variables.

Variable	Unit	Mean	Std. Dev.	Min	Max	Obs
ESO_2_	Hundred thousand tons	48.880	37.676	0.088	162.864	390
ENO_2_	Hundred thousand tons	39.406	30.033	0.801	127.360	390
GSO_2_	Ton/hundred million yuan	50.305	52.745	0.036	369.923	390
GNO_2_	Ton/hundred million yuan	35.964	34.208	0.33	291.191	390
BET	%	17.160	6.096	4.237	43.905	390
TIUP	Ten thousand piece	2.869	4.476	0.011	34.248	390
PGDP	Ten thousand yuan/person	3.617	1.902	0.692	11.261	390
ISR	/	8.094	9.706	1.289	59.186	390
ISU	%	108.244	62.194	49.959	516.924	390
PCP	SQM/person	11.957	2.966	5.89	21.049	390
FSS	%	44.703	17.633	12.113	92.291	390

Note: Std. Dev. denotes standard deviation; Obs denotes observations.

**Table 3:** Model specification test results.

	Threshold Type	(1)	(2)	(3)	(4)
		SO_2_	NO_2_	GSO_2_	GNO_2_
Estimator of PGDP threshold	Single threshold	3.116	3.116	1.782	3.116
	Double threshold	3.116	3.056	1.759	3.116
		8.689	4.476	3.056	0.958
	Triple threshold	2.019	6.349	0.958	8.688
F test of UR threshold effect	Single threshold	49.07 ***	37.33 **	120.97 *	26.31 *
	Double threshold	21.88	9.91	42.85 *	28.93 *
	Triple threshold	9.52	9.78	42.98	8.01
*p* value	Single threshold	(0.010)	(0.050)	(0.000)	(0.087)

Note: *p*-Values in parentheses, * *p* < 0.1, ** *p* < 0.05, *** *p* < 0.001.

**Table 4:** Threshold regression results.

	(1)	(2)	(3)	(4)	(5)	(6)	(7)	(8)
	SO_2_	SO_2_	NO_2_	NO_2_	GSO_2_	GSO_2_	GNO_2_	GNO_2_
BET (PGDP ≤ δ)	−1.082 **	−0.840 **	−0.559 *	−0.614 **	−1.863 ***	−1.568 **	−2.450 ***	−1.982 ***
	(0.007)	(0.041)	(0.051)	(0.043)	(0.001)	(0.002)	(0.000)	(0.000)
BET (PGDP > δ)	−2.125 ***	−1.832 ***	−1.204 ***	−1.259 ***	−4.603 ***	−3.248 ***	−3.241 ***	−2.741 ***
	(0.000)	(0.000)	(0.000)	(0.000)	(0.000)	(0.000)	(0.000)	(0.000)
TIUP	−4.012 ***	−3.813 ***	−3.291 ***	−3.251 ***	1.151 **	1.494 **	−0.445	−0.465
	(0.000)	(0.000)	(0.000)	(0.000)	(0.048)	(0.004)	(0.346)	(0.321)
PGDP	3.581 **	4.971 **	7.872 ***	7.382 ***	−12.684 ***	−4.505 *	−0.877	2.822
	(0.043)	(0.013)	(0.000)	(0.000)	(0.000)	(0.060)	(0.648)	(0.209)
ISU	−0.281 ***	−0.234 ***	−0.255 ***	−0.249 ***	−0.255 ***	−0.156 **	−0.227 ***	−0.202 ***
	(0.000)	(0.000)	(0.000)	(0.000)	(0.000)	(0.001)	(0.000)	(0.000)
PCP		−2.608 ***		0.097		−9.036 ***		−2.938 ***
		(0.000)		(0.850)		(0.000)		(0.000)
FSS		0.776 ***		0.146		0.529 ^**^		0.118
		(0.001)		(0.372)		(0.046)		(0.621)
cons	104.657 ***	86.004 ***	62.662 ***	57.030 ***	193.789 ***	215.678 ***	113.341 ***	118.915 ***
	(0.000)	(0.000)	(0.000)	(0.000)	(0.000)	(0.000)	(0.000)	(0.000)
N	390.000	390.000	390.000	390.000	390.000	390.000	390.000	390.000
R^2^	0.536	0.565	0.454	0.452	0.629	0.711	0.382	0.405
F	96.478	77.355	71.404	50.955	138.744	141.782	54.946	42.922

Note: *p*-Values in parentheses, * *p* < 0.1, ** *p* < 0.05, *** *p* < 0.001.

**Table 5:** Mediating effect on SO_2_ and NO_2_.

	SO_2_				NO_2_			
	c	ab	$c^{\prime }$	ab/c	c	ab	$c^{\prime }$	ab/c
BET	−3.188 **				−1.403 **			
	(0.001)				(0.018)			
TIUP		−0.389 ***	−2.799 ***	0.122		−0.238 ***	−1.165 ***	0.170
		(0.000)	(0.000)			(0.007)	(0.002)	
PGDP		−1.892 ***	−1.296 **	0.593		−0.152 ***	−1.251 **	0.108
		(0.000)	(0.028)			(0.004)	(0.000)	
ISU		−1.130 ***	−2.057 ***	0.354		−0.911 ***	−0.493	0.649
		(0.000)	(0.000)			(0.000)	(0.280)	

Note: *p*-Values in parentheses, ** *p* < 0.05, *** *p* < 0.001.

**Table 6:** Mediating effect on GSO_2_ and GNO_2_.

	GSO_2_				GNO_2_			
	c	ab	$c^{\prime }$	ab/c	c	ab	$c^{\prime }$	ab/c
BET	−7.858 ***				−4.328 ***			
	(0.000)				(0.000)			
TIUP		−0.245 **	−7.613 ***	0.031		−0.155 **	−4.173 ***	0.036
		(0.011)	(0.000)			(0.011)	(0.000)	
PGDP		−2.434 ***	−5.424 ***	0.310		−1.112 ***	−3.216 ***	0.257
		(0.000)	(0.000)			(0.000)	(0.000)	
ISU		−1.172 ***	−6.687 ***	0.149		−0.752 ***	−3.576 ***	0.174
		(0.000)	(0.000)			(0.000)	(0.000)	

Note: *p*-Values in parentheses, ** *p* < 0.05, *** *p* < 0.001.

## Data Availability

Publicly archived datasets China Statistical Yearbook on Environment and China Statistical Yearbook 2009–2018 were analyzed in this study. These data can be found at: https://data.cnki.net (accessed on 12 March 2022). Other raw/processed data required to reproduce these findings cannot be shared at this time, as the data also form part of an ongoing study.
